# Numerical Simulation of the Impact of Water Vapour and Moisture Blockers in Energy Diagnostics of Ventilated Partitions

**DOI:** 10.3390/ma15228257

**Published:** 2022-11-21

**Authors:** Barbara Ksit, Anna Szymczak-Graczyk, Roman Pilch

**Affiliations:** 1 Institute of Building Engineering, Faculty of Civil and Transport Engineering, Poznan University of Technology, Piotrowo 5, 60-965 Poznań, Poland; 2 Department of Construction and Geoengineering, Faculty of Environmental and Mechanical Engineering, Poznan University of Life Sciences, Piątkowska 94E, 60-649 Poznań, Poland; 3 Department of Architecture and Town Planning, Faculty of Civil, Environmental Engineering and Architecture, Bydgoszcz University of Science and Technology, Prof. S. Kaliskiego 7, 85-796 Bydgoszcz, Poland

**Keywords:** water vapour, operational moisture, numerical analysis, dew point, flexible waterproofing materials

## Abstract

Current trends towards saving energy and designing sustainable buildings result in most designers focusing on achieving the best thermal parameters, thereby neglecting a careful moisture analysis. Excessive moisture content in building partitions degrades the mechanical properties of materials, reduces thermal insulation properties (which leads to an increase in the demand for thermal energy) and worsens the microclimate in rooms. Modern digital solutions help create appropriate models of partitions that work for many years in good environmental conditions. According to the analysis of air parameters, 1 m^3^ of air at 20 °C contains approx. 17.3 g of water. When the temperature of the air reaches the dew point temperature, water vapour condenses. The dew point depends on air temperature and relative air humidity; for instance, at the same air temperature of 20 °C, the dew point temperature at 40% relative air humidity is 6 °C, whereas at 90% relative humidity, it is over 18 °C. This means that the higher the value of relative humidity in the room at a certain temperature, the lower the temperature that will cause condensation. The article presents a numerical analysis of the insulation work of flexible materials within the layers of ventilated partitions in an 8-year simulated period of varying environmental conditions. The aim of the article is to analyze different models and variants of ventilated partition operation with respect to the advisability of using a vapour barrier to avoid the problem of destruction of thermal insulation and finishing layers of a ventilated roof.

## 1. Introduction

Water is one of the most important substances for humans, being the source of life, yet it is also the cause of degradation of building partitions. In various forms, it accompanies people every day and wherever they are [1,2,3,4,5,6,7]. Renovation processes carried out in multi-family buildings that are subject to municipal management or the exploitation of residential buildings located in areas under conservation protection, where tenants have a varying level of construction knowledge, sometimes contribute to an increase in moisture content in building partitions. Various phenomena observed by researchers lead to an increase in moisture content in walls and ceilings: these include an insufficient number of gravity ventilation chimneys in buildings subject to multiple functional reconstructions; or replacement of window frames (usually wooden) with new ones that no longer allow free air migration and removal of water vapour from rooms. The operating moisture is the water vapour generated by residents and their activities inside a building. Various individual operating habits are usually the key reason for the occurrence of extreme hygrothermal conditions in buildings, which can often significantly exceed the ones specified in building standards [8,9,10]. The limit value for relative air humidity at the surface of a building is 80%. With ambient air at a temperature of 20 °C and 50% relative humidity, the allowable external wall surface temperature with reference to the protection against fungal coverage is 12.6 °C. The limit value of the dew point temperature on the surface of an internal partition (the temperature at the state of saturation with water vapour, meaning relative air humidity equal to 100%) at 50% relative air humidity is 9.3 °C and the saturated water vapour pressure is 23.40 hPa. As shown in the publications, condensation on building partitions predominantly appears near to critical thermal places, when the internal temperature of external surfaces drops below the dew point [11,12,13,14,15,16,17]. The condensate produced in this way contributes to the development of mould fungi, which degrade both living organisms and building elements. The spores produced by their fruiting bodies circulate in the air and are inhaled by people, which make them feel unwell or tired. Also, they may contribute to the development of various diseases or even death. In addition, mould reduces the strength and durability of elements and structures, and the resultant moisture negatively affects the technical parameters of materials and increases the risk of a building catastrophe [5,6,18,19,20,21,22].

Condensation can also occur inside partitions. The transport of water vapour into a partition takes place through the process of diffusion. The course of diffusion depends on the coefficients of water vapour transmission in the materials the partition is made of [15,23,24]. They determine the amount of water vapour, measured in kilograms, which can penetrate through 1 m^2^ of a 1-metre thick material in 1 s with a pressure difference between the two sides of the material equal to 1 Pa. On the basis of the amount of the air removed from individual rooms indicated by the standards [25,26], it is possible to estimate the required multiplicity of air exchange inside, which determines how many times the air is changed per hour. For single-family houses, the value of this indicator ranges from 0.5 to 0.8 or even 1.0. The index 1.0 means that all the air in the room will be replaced within one hour. Movement of the air causes a change in heat exchange through convection between a person and their environment, which affects the individual feeling of comfort. Receptors in the skin immediately receive information about the thermal environment in which a human body is located, but when the skin is cooled to approx. 32 °C, it begins to feel cold, and after exceeding 37 °C, it begins to sweat [27,28,29]. Too low an air velocity in the room prevents proper heat dissipation from a human body, which results in general heat sensation. On the other hand, when the air is exchanged too fast, more heat is given off, which means that a person starts to feel cold. At the comfort temperature, which is from 18 to 21 °C, people do not feel the effect of relative air humidity when it is in the range of 30–60% [28,29,30,31,32,33,34,35].

The basic source of moisture content in buildings is the emission of water vapour related to metabolic changes occurring in their residents and from the activities they carry out inside.

On the subject of diffusion and condensation of moisture in the layers of the building envelope, the literature mandates waterproof layers. Professional practice shows that such layers are standardly adopted by architects for all roof constructions and especially for ventilated roofs. Using the possibilities afforded by numerical analysis, simulation models with different variants of waterproof barrier placement were elaborated for this paper. The operation of such a barrier and the condition of the partition were analyzed over the 8 years of a partition’s operation.

The aim of the study was to show that double-sided flat roofs containing air spaces should be treated differently than pitched roofs containing small gaps in the structure of their layers, acting as ventilated spaces. The durability of insulating materials as well as the reliability of their characteristics and parameters depends to a large extent on the environment in which they work. Moreover, deteriorating insulation properties of partitions may lead to degradation and, consequently, to an even greater reduction in their thermal efficiency. Change in the technical parameters of insulating materials caused by excessive moisture sorption over time can involve introducing additional security measures to ensure the safe use of buildings. In extreme cases, it may require serious structural changes or even the repurposing of the the facilities and how they are used. In the case of flat roofs, it is not always necessary to use flexible waterproofing materials in ventilated partitions to ensure the protection of thermal insulation against moisture. The current paper shows that it is worth using multi-variant modelling of building partitions to reduce the energy consumed for heating and to prevent insulating materials from absorbing moisture. The paper presents the results of numerical simulations over the years, forming the basis for our conclusion, namely, that construction solutions that are currently very popular cannot be universally used for all types of roofs. An important aspect of using or not using flexible waterproofing materials is the method of ventilating the space present in the layers of a partition. The results presented in the paper demonstrate the legitimacy of solutions that are less expensive and easier to implement.

## 2. Materials and Methods

### 2.1. Operational Concerns Regarding Water Vapour Generation Inside Buildings

The main sources of water vapour in buildings include technological moisture, increasing relative air humidity in the first years after the construction, and operational moisture [9,36]. The people who use the building are themselves the constant generators of a large amount of water vapour. As a result of metabolic changes, humans release much heat, which is emitted to the environment through radiation, convection, and evaporation. Summing up the percentage results of heat dissipation through radiation and convection, 79% of the share is called dry heat (sensible heat), the remaining 21% of heat given off by water evaporation and breathing is called moist heat (latent heat) [24,29,37].

However, a larger amount of water vapour is generated by various activities performed by people, and it has the greatest and fastest impact on the value of relative humidity of the indoor air [10]. On average, it is assumed that the amount of water released by a family of four in metabolic processes is 0.21 L/h i.e., 5 L/day, or 1.25 L/day per person [38]. For two adults, the average emission of water vapour is 6.5 kg/day, and for parents with two children—10.9 kg/day. For one person the emission is assumed to be 4.4 kg/day with the standard deviation 1.73 kg [39]. There is one more source of moisture in the room, which is houseplants that need to be watered regularly. Almost all the water that is provided to plants evaporates, as only 0.2% of it is required for vegetation growth. 5 to 7 potted plants can release approx. 0.5 L of water in 24 h [40,41]. Water vapour is a variable component of the atmospheric air and comes primarily from the process of evaporation of water from the earth’s surface and precipitation. The amount of water vapour in a unit of air equal to 1 m^3^ decreases or increases depending on surrounding environmental conditions, and its value, referred to as absolute humidity, is expressed in g/m^3^ [4,42]. Water vapour content in the air is limited and depends on air temperature. The warmer it is, the more water vapour it can contain. The maximum possible value of filling the air with water vapour is defined as the state of saturation. Exact values of water vapour content in the air with the maximum humidity depending on temperature are compiled in Table 1.

**Figure 1:**
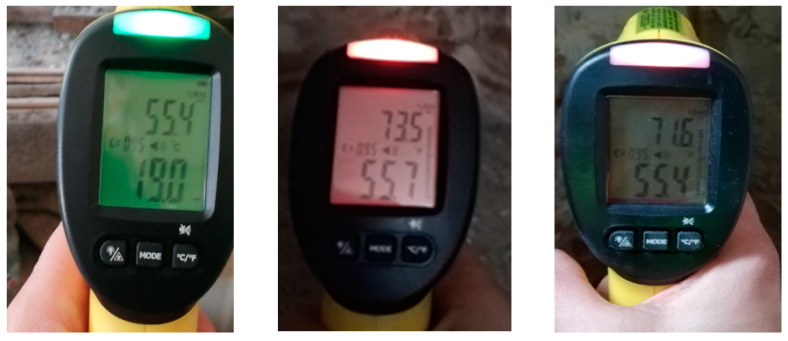
Examples of air humidity and air temperature tests using a pyrometer and a dew point scanner.

In diagnostics assessing existing building solutions, non-invasive moisture meters (pyrometer Trotec BP25, Heinsberg, Germany) are used, which provide the percentage of relative humidity in analysed rooms (Figure 1).

In situ and digital analyses take into account the value of relative humidity i.e., a percentage measure of water vapour content in the air. It is expressed by the ratio of the actual water vapour pressure contained in the air to the maximum water vapour pressure possible at a given temperature (saturated vapour pressure) [4,6,10]. When using the concept of relative humidity, it is always required to provide the air temperature at which it is measured, since the percentage alone says nothing about the actual water vapour content. Research [43] shows that the actual moisture content in the air will remain the same, whereas the saturation level will change: i.e., at 10 °C the value of relative humidity is 100% and at 30 °C it is 28%. In order to eliminate water vapour from rooms, appropriate ventilation systems should be designed.

In the case of gravity ventilation, the occurrence of moisture amplitudes inside rooms is inevitable. To reduce the risk of moisture occurrence in external partitions, ventilation gaps are used [36,44,45,46,47,48,49]. The dimensions of ventilation gaps must comply with [50] and with the information provided in [51]. Other related standards [52,53] provide parameters that should also be considered in order to correctly model building partitions. Double protection of heterogeneous partitions, with two layers of gaps, is used by introducing an obstacle preventing water vapour from moving from inside a room to the outside, in the form of a layer which prevents its penetration of the partitionThe Polish climate is more hazardous than many for building partitions due to the large amplitude of temperature difference, increasing the number of cycles of condensation formation especially in roofs. As this phenomenon carries the risk of thermal insulation materials becoming damp, it is necessary to minimize the potential damage. Because water vapour in a building penetrates its roof the fastest, in order to ensure this partition provides its protective work, it is most often shaped as a multi-layer structure constructed with the use of flexible waterproofing materials [54,55,56].

The minimum cross-sections of ventilation gaps in inclined partitions at a roof slope of ≥5° and <5° are specified in Tables [50], which are the recommendations of the Association of German Roofers. As early as 1997, the guidelines for roofs with a slope of ≥10% [54,55,56] recommended that the total diffusion resistance should increase with an increase in the length of the ventilating air path, i.e., with an increase in the length of rafters.

A double ventilation gap is required particularly when the roofing is laid on a board (including full boarding), or when a foil with low vapour permeability is used as the initial covering layer (protecting against the penetration of moisture from the outside, but at the same time hindering the diffusion of water vapour from the inside to the outside), and the roofing itself is laid on patches and counter-patches. In addition, it is recommended for roofs with very complex shapes. In such cases, it is necessary to provide separate ventilation for patches and for the main roofing. One ventilation gap should be designed between the thermal insulation material and the layer of initial covering used in a given design (board, full boarding with tar paper, low vapour permeability membrane). In this way, the thermal insulation against moisture is protected (the joint removes the moisture that has penetrated through the vapour barrier to the insulation). It should be a few centimetres wide, with air inlet openings (under the eaves, in the soffit) and outlet openings (under the ridge, ventilation grates in the gable walls). The second gap must be between the initial covering layer and the actual roof covering, thanks to which the roof finishing materials dry faster [45,46,47,48,49].

Due to the various properties of materials related to the transmission of water vapour, the arrangement of layers in external partitions should be carefully selected.

The knowledge of the water vapour permeability coefficient δ or the thickness of the equivalent air layer S_d_ is needed to calculate the water vapour permeability of the material with thickness d [57,58,59] calculated with Formula (1):

(1) $$W_{p}=\frac{\;\delta }{d}=\frac{\delta_{0}}{\mu \cdot d}=\frac{2\cdot 10^{-4}}{S_{d\;}}$$

where:

W_p_—water vapour permeability $\left[\frac{\mathrm{kg}}{m^{2}\cdot s\cdot \mathrm{Pa}}\right]$
δ—water vapour permeability coefficient, $\left[\frac{\mathrm{kg}}{m\cdot s\cdot \mathrm{Pa}}\right]$
d—material layer thickness, [m]
δ_0_—air vapour permeability (δ_0_ = 2× 10^−4^), $\left[\frac{\mathrm{kg}}{m\cdot s\cdot \mathrm{Pa}}\right]$
µ—diffusion resistance coefficient, [unitless]
Sd—thickness of the equivalent air layer, [m]

The diffusion resistance Z_p_ is the reciprocal of water vapour permeability and describes the resistance of a building element to water vapour diffusion, according to Formula (2).

(2) $$Z_{p}=\frac{\;1}{W_{p}}=\frac{\;d}{\;\delta }=\frac{S_{d}}{2\cdot 10^{-4\;}}$$

In order to determine the value of diffusion resistance of a multilayer element, the values of diffusion resistance of individual material layers should be summed up, as shown by Formula (3):

(3) $$Z_{p}=\underset{n}{\sum }\frac{S_{d}}{2\cdot 10^{-4\;}}$$

where n is the individual layer of material.

The layers should be arranged in such a manner as to allow free flow of water vapour through the partition and to avoid condensation of water vapour inside it. Table 2 below shows the simulations of the amount of water vapour reaching the attic in 24 h.

Assuming a model building with a cubature of 420 m^3^, the air flow is from 4.2 to 10.5 thousand m^3^. Thus, in 2940 m^3^, there is 40,689.6 g of water vapour, and in 5250 m^3^—72,660 g. After adding the appropriate amount of moisture generated in the building, in total, in 2940 m^3^, there is 53,818.6 g of water vapour; and in 5250 m^3^, as much as 84,689 g (Table 3).

Dividing the obtained amount of water vapour by the volume of air reaching the attic, we obtain the absolute humidity values equal to 18.31 g/m^3^ for 10-fold air exchange and 16.13 g/m^3^ for 25-fold air exchange.

For 10-fold exchange, we obtain the state of air supersaturation, as the maximum amount of water vapour that the air can hold, according to the guidelines, is 17.3 g/m^3^. This result indicates that the value of relative humidity in the rooms is 100%, and 1 g of water drops out of the building partitions from each 1 m^3^.

In the case of 25-fold air exchange, the value of relative humidity is at the level of 93% (Table 4) and the amount of water condensed from 1 m^3^ of air will need to be calculated.

In the design of external partitions, due to the diffusion of water vapour, the principle of arranging the layers according to their decreasing diffusion resistance from the inside to the outside is applied [11,12]. As a result, it is more difficult for water vapour flowing through the partition to reach the state of saturation, despite ever decreasing temperature, so that the increased condensation of water vapour inside the partition does not occur. It is important to carry out adequate air ventilation in a given area. Ventilation openings should be designed so that they ensure the required air exchange in the flat roof space and are not blocked by thermal insulation materials.

**Figure 2:**
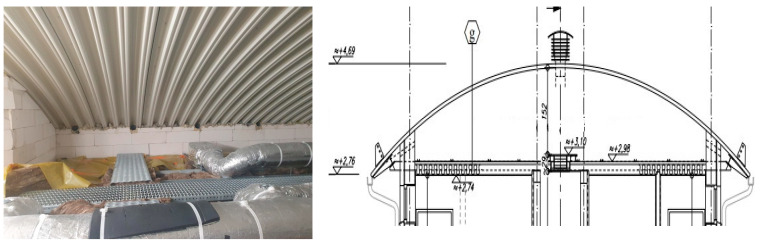
Geometrically complicated roof space in a ventilated roof (analysed here).

Designing partitions according to the above-mentioned principle is not always possible, however. Indeed as a rule, it is necessary to analyse the functioning of a particular partition in terms of humidity. Figure 2 shows an example of a geometrically complex roof space in a ventilated roof, analysed here for this article.

### 2.2. Flexible Waterproofing Materials Used in Ventilated Partitions

These materials are used to protect partitions against water penetration. The division of materials and their names are presented in Table 5 in accordance with the list contained in the Glossary of Roofing Terms and Names [57].

Depending on polymers contained in processed plastics, foils can be divided into thermoplastic (plastomeric) and elastomeric.

Initial covering foils are materials with low vapour-permeability, and are the first plastic products that replaced roof boarding and tar paper over 60 years ago. A roof in which ICF will be used should have two ventilation gaps: one under the covering, and the other with a sublayer of foil. These types of foils are also often called vapour permeable foils.

Initial covering membranes are foils with high vapour-permeability, with S_d_ < 0.1 m (optimally from 0.015 to 0.045 m), and are currently the most common material used for the initial covering of pitched roofs.

In most cases, roof membranes are made of non-woven polypropylene, which is highly vapour-permeable buthas little resistance to the water column, and therefore must be additionally provided with a delicate film, i.e., a functional film and non-woven polypropylene fleece.

Among initial covering membranes, roof screens with increased durability, grammage and strength deserve a special distinction [60,61,62,63,64,65,66,67].

The market of construction materials offers diffusion-active foils, the work of which is to protect structures both in summer and winter. In winter, the average humidity of the vapour barrier environment is approx. 40%. Diffusion is directed outward from the heated interior. The vapour barrier should be highly vapour-tight during this period in order to protect structures from condensation. In summer, the average ambient humidity of the vapour barrier is approx. 80%, and the diffusion flow is reversed. During this period, the vapour barrier should be able to become permeable so as to allow moisture to dry out.

It should also be noted that the vapour barrier foil, which is used in flat roofs with an increased concentration of water vapour (e.g., in rooms with showers), should not be placed on thermal materials. It is also not advisable to wrap the contact points between materials such as between wool and external wall. Moreover, the foil should be attached to the external wall as required, turning it downwards with a slight drip upwards.

### 2.3. Case Study

In order to analyse the moisture flow of water vapour, the authors modelled a barrier consisting of the following materials: acrylic paint inside, plasterboard, PE foil, mineral wool, glass mineral wool, air space with variable height from 20 to 80 cm, and trapezoidal sheet (Figure 2).

For such a complex case, models based on a system of non-linear partial differential equations describing the non-stationary, coupled transport of heat and moisture in building materials and partitions were used [68]. Simulation calculations were made using the WUFI PRO 6.5 software, used for a one-dimensional analysis of non-stationary processes of heat and moisture flow through building partitions. The software was developed at the Fraunhofer Institute for Building Physics in Holzkirchen (Germany). The simulation calculations included, among others:

− variable properties of the material depending on humidity and temperature;
− additional thermal transport processes, such as latent heat transport by water vapour flows;
− additional heat sources due to solar radiation;
− parameters dependent on the environmental conditions, such as wind and rain action.

In the calculations performed, the parameters of the external climate were adopted on the basis of a “typical meteorological year” included in the software.

Natural gravity ventilation was adopted for the analyses, and the foil parameters took into account possible leaks resulting from the perforation of the foil with mechanical fasteners used for attaching plasterboards or for fixing lamps. A high level of internal air humidity was adopted, and the calculation variants assumed an 8-year simulated period of environmental actions. This is long enough to show a possible increase in moisture content in built-in materials and occurrence of conditions for the development of biological aggression. The transport of moisture in the partition was assumed to be two-way.

### 2.4. Calculation Variants

Various arrangements were considered, relative to the polyethylene foil applied or its absence, and to ventilation of the space above the thermal insulation materials used. The technical data sheets provided by the manufacturers lacked the information specifying both the material density and diffusion resistance parameters. Therefore, on the basis of their technical knowledgeand after considering various options, the authors chose the most realistic parameters for the declared materials, as adapted to the technological processes taking place during execution and operation.

**Figure 3:**
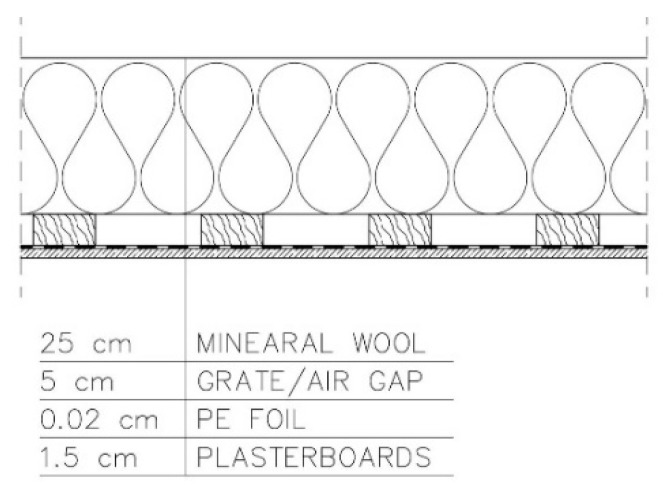
Variant 1: PE foil under the grate for fixing plasterboards. Roof ventilation equal to 20 changes per hour.

**Figure 4:**
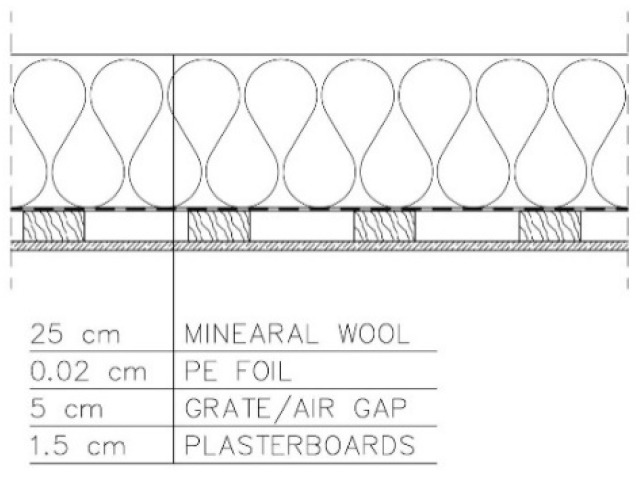
Variant 2: PE foil above the grate for fixing plasterboards. Roof ventilation equal to 20 changes per hour.

**Figure 5:**
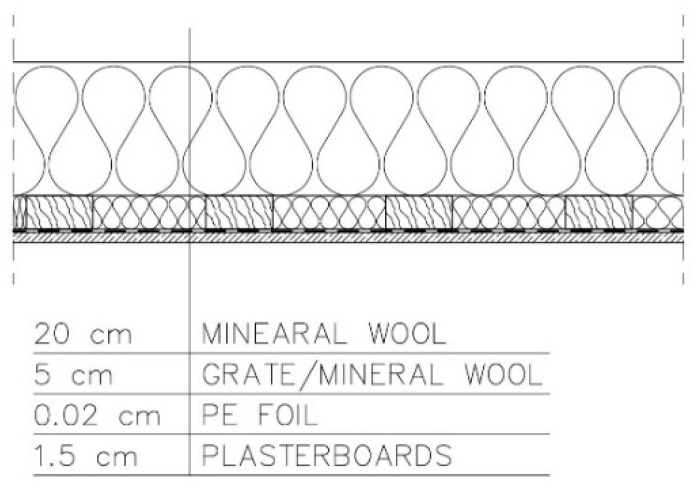
Variant 3: PE foil under the grate for fixing plasterboards. Mineral wool fills the air gap formed by the grate. Roof ventilation equal to 20 changes per hour.

**Figure 6:**
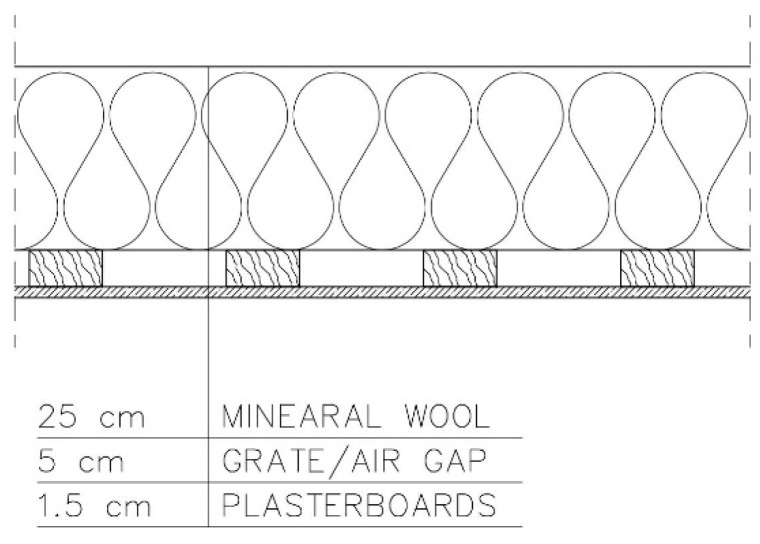
Variant 4: no PE foil in the arrangement of layers. Roof ventilation equal to 20 changes per hour.

**Figure 7:**
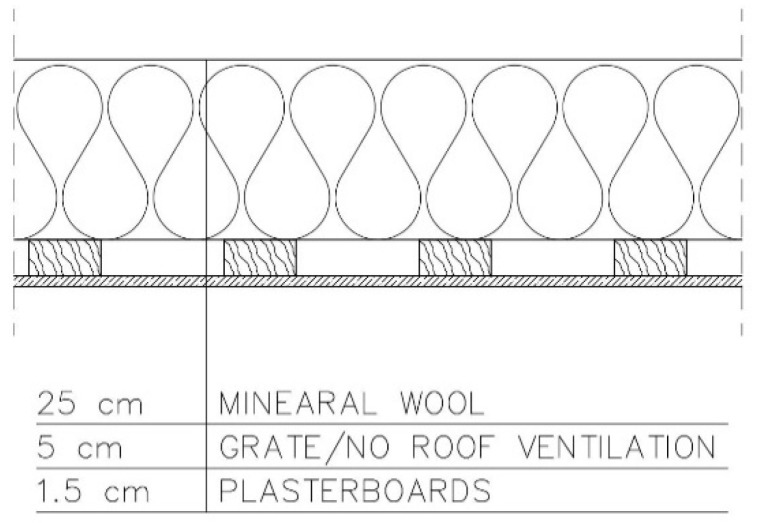
Variant 5: no PE foil in the arrangement of layers, no roof ventilation.

The analysed variants are presented below:

− Variant 1—PE foil under the grate for fixing plasterboards. Roof ventilation equal to 20 changes per hour (Figure 3).

− Variant 2—PE foil above the grate for fixing plasterboards. Roof ventilation equal to 20 changes per hour (Figure 4).

− Variant 3—PE foil under the grate for fixing plasterboards. Mineral wool fills the air gap formed by the grate. Roof ventilation equal to 20 changes per hour (Figure 5).

− Variant 4—no PE foil in the arrangement of layers. Roof ventilation equal to 20 changes per hour (Figure 6).

− Variant 5—no PE foil in the arrangement of layers. No roof ventilation (Figure 7).

## 3. Results

**Figure 8:**
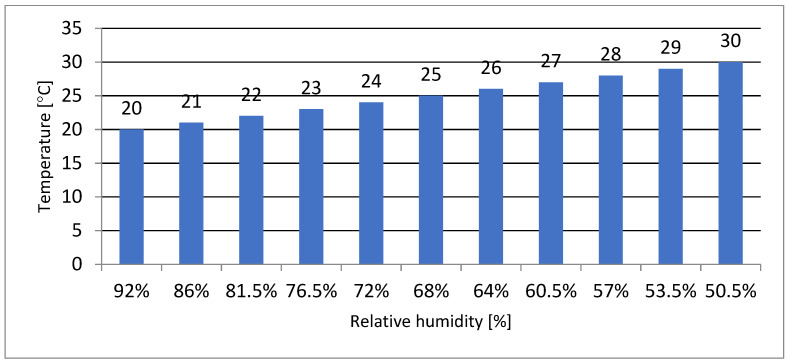
Dependence of temperature [°C] on relative humidity [4].

The calculations are summarized below, assuming the value of water vapour pressure 2150 Pa and the corresponding values of temperature and relative humidity (Figure 8).

The highest temperature was 25 °C and corresponded to 70% relative humidity. The corresponding partial pressure of water vapour was 2217 Pa. For individual calculation variants from the data cloud, there are shown changes in moisture content in thermal insulation materials and in plasterboard. In addition, an isopleth of the internal surface is shown (interpretation based on the WUFI software). The WUFI program divides materials into:

− LIM B I: bio-utilizable substrate, i.e., wallpaper, plasterboard, products made from easily degradable material, material for permanently elastic joints, etc.;
− LIM B II: substrates with porous structure, i.e., plasters, mineral building materials, some types of wood, insulating materials not belonging to group I, etc. If strongly contaminated, these materials belong to group I [69].

On the isopleth map, the material curves developed according to the guidelines of the numerical program LIM B I and LIM B II—below which mould development is not normally expected—express the limits for building materials or for the conditions for their incorporation into buildings.

The colour of each point on the isopleth indicates when that point occurred during the computation. The diagrams show the changes in moisture content of individual materials over eight years for variants 1–5. Moisture content [kg/m^3^] is marked on the vertical axis and the time period from 01 January 2023 to 01 January 2031 was simulated on the horizontal axis. The isopleth diagrams of the internal surface of the partition show the relationship between relative humidity [%] and temperature for variants 1–5 [70].

**Figure 9:**
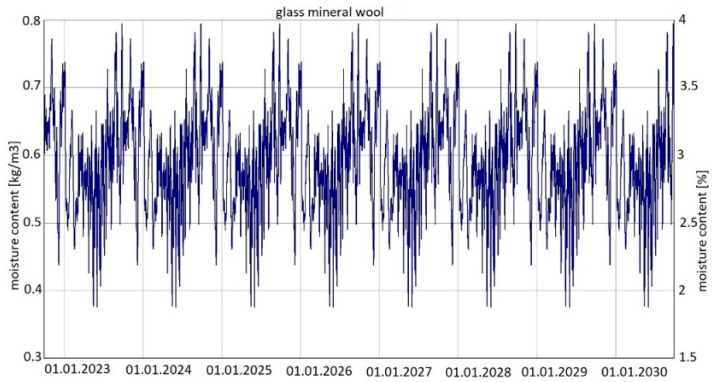
Changes in moisture content in glass mineral wool over eight years, variants 1–3 [70].

**Figure 10:**
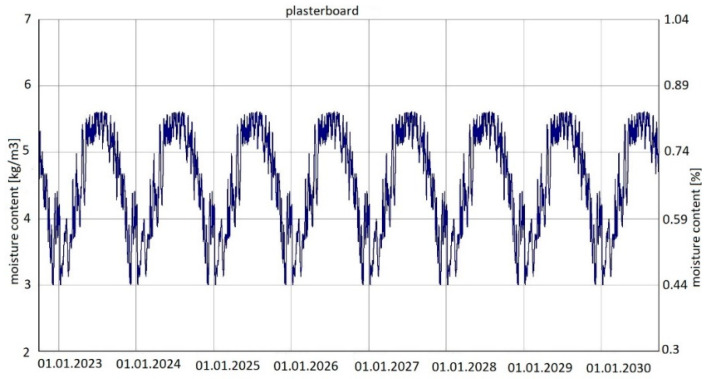
Changes in moisture content in plasterboard over eight years, variants 1–3 [70].

For variants 1, 2 and 3, the same analysis results were found for changes in moisture content in both wool and plasterboard. Figure 9 shows the changes in moisture content of glass mineral wool over the analysed period of eight years, while Figure 10 shows the changes in moisture content of plasterboard over the same period.

**Figure 11:**
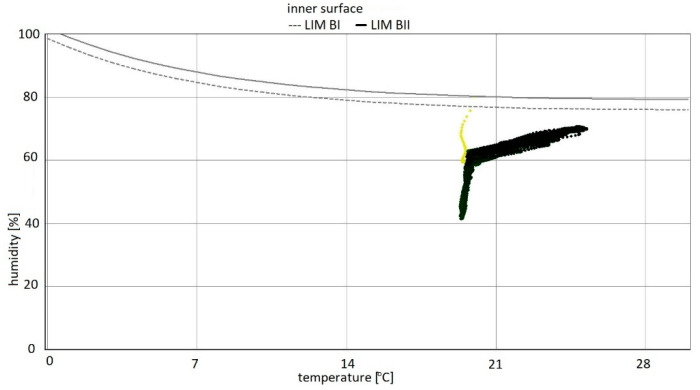
Isopleth of the inner surface of the partition, variants 1–3 [70].

For variants 1–3 (Figure 3, Figure 4 and Figure 5), the moisture level for mineral wool during the analysed period ranged from 1.7% to 4%, and for plasterboard from 0.44% to 0.80%. For these same variants 1–3, the humidity—temperature isopleth is presented in Figure 11.

The isopleths of the LIM B I (dashed line) and LIM B II (solid line) curves, i.e., contour charts of the mould growth rate as a function of temperature (horizontal axis) and humidity (vertical axis) as shown in the diagram, do not intersect with the area of points for the analysed partition.

Mould spots appeared at a minimum relative humidity of 41% and a temperature of 18 °C. In the isopleth (Figure 10), there is no critical value, i.e., no point of intersection of the curves with the area of points.

**Figure 12:**
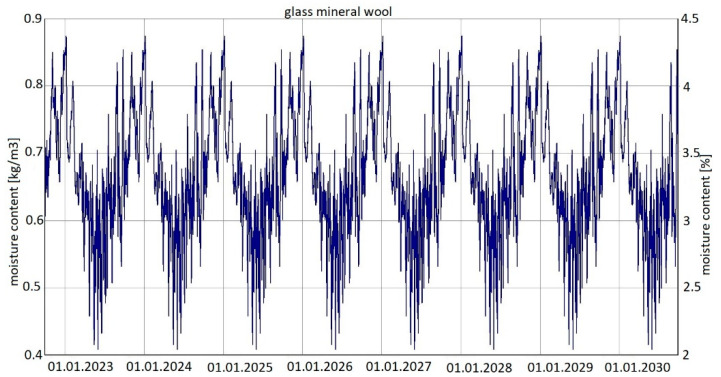
Changes in moisture content in glass mineral wool over eight years, variant 4 [70].

**Figure 13:**
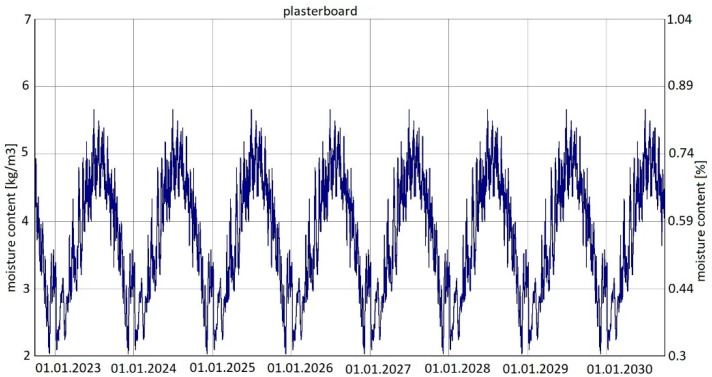
Changes in moisture content in plasterboard over eight years, variant 4 [70].

**Figure 14:**
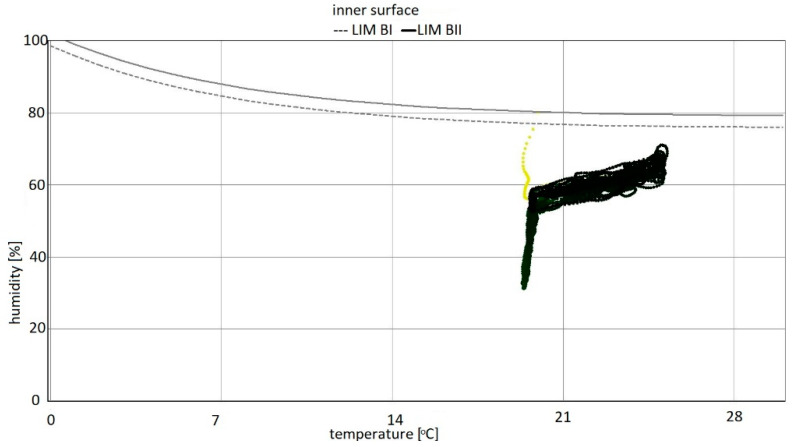
Isopleth of the inner surface of the partition, variant 4 [70].

For variant 4 (Figure 6), moisture content depending on the season is shown in the diagrams for mineral wool (Figure 12) and for plasterboard (Figure 13), while the relative humidity—temperature isopleth is shown in Figure 14.

The moisture level for mineral wool during the analysed period ranged from 2.1% to 4.7%; and for plasterboard, depending on the season, it ranged from 0.3% to 0.85%.

As previously, the isopleths of the LIM B I (dashed line) and LIM B II (solid line) curves, i.e., contour charts of the mould growth rate as a function of temperature (horizontal axis) and humidity (vertical axis) appearing in the diagram, do not intersect with the area of points for the analysed partition. Mould spots appeared at a minimum relative humidity of 30% and a temperature of 18 °C. There is no critical value in the isopleth (Figure 14), i.e., no point where the curves intersect with the area of points.

**Figure 15:**
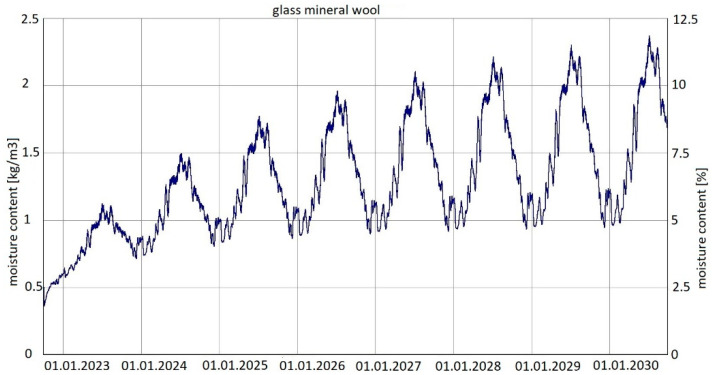
Changes in moisture content in glass mineral wool over eight years, variant 5 [70].

**Figure 16:**
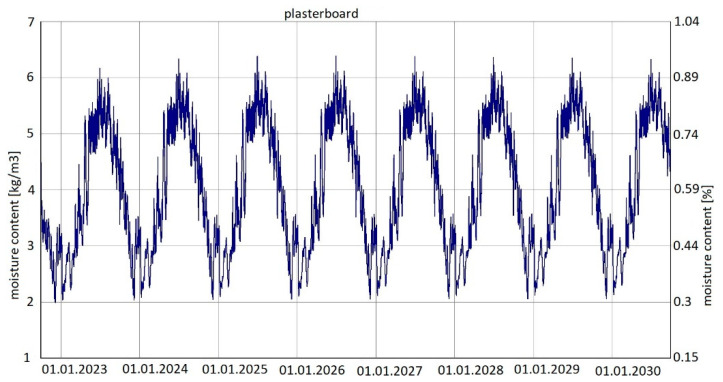
Changes in moisture content in plasterboard over eight years, variant 5 [70].

**Figure 17:**
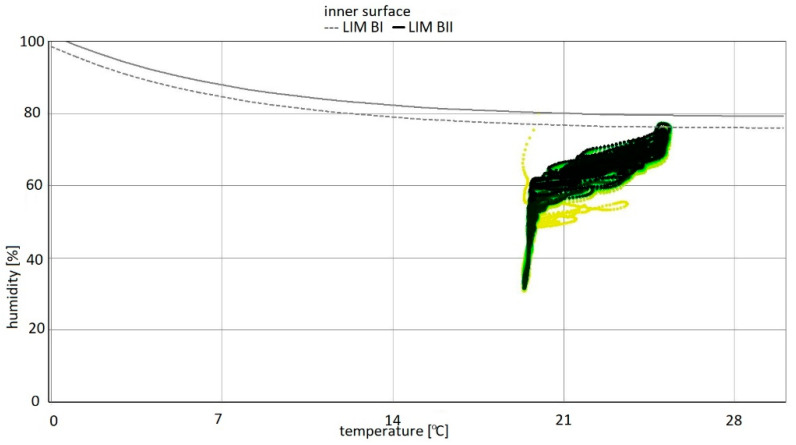
Isopleth of the inner surface of the partition, variant 5 [70].

For the last of the analysed variants, variant 5 (Figure 7), moisture content depending on the season is shown in the diagrams for mineral wool (Figure 15) and for plasterboard (Figure 16), while the relative humidity—temperature isopleth is presented in Figure 17.

Moisture content in variant 5 depending on the season shown in the diagram (Figure 15) for mineral wool increased throughout the research period, starting from 2% in winter to 12% (maximum value last year in summer). The maximum value was approx. 2.4 kg/m^3^.

Moisture content in variant 5 depending on the season shown in the diagram (Figure 16) for plasterboard fluctuated throughout the research period from 0.3% in winter to 0.92% (maximum value last year in summer). The maximum value was approx. 6.1 kg/m^3^.

The isopleths of the LIM B I (dashed line) and LIM B II (solid line) curves i.e., contour charts of the mould growth rate as a function of temperature (horizontal axis) and humidity (vertical axis), do intersect with the area of points for the partition analysed. The risk of mould development appeared at a minimum relative humidity of 78% and a temperature of approx. 24 °C (Figure 17).

By analysing the isopleths for variants 1–5 (Figure 11, Figure 14 and Figure 17), it can be stated that the points from yellow to black show the situation on the inner surface during the simulation period. The coordinates of each point are determined by temperature and relative humidity. If the point is located above the LIM boundary curve as in variant 5, then mould development can be expected; whereas if below the limit as in variants 1–4, then no biological aggression is to be expected. As the analysis showed, the conditions conducive to the development of mould aggression occurred when the flat roof ceased to be well ventilated. The mere arrangement of the moisture blocking layer is irrelevant in variants 1–3.

Summarizing, it can be concluded that the results shown in the Figures regarding thermal insulation materials and plasterboards clearly indicate the trend of fluctuating results depending on the season. Figure 9, Figure 10, Figure 12, Figure 13, Figure 15 and Figure 16 indicate changes in moisture content in thermal insulation materials (Figure 9, Figure 12 and Figure 15) and in plasterboard (Figure 10, Figure 13 and Figure 16), while isopleths (Figure 11, Figure 14 and Figure 17) indicate the possibility of mould development on the inner surface of the material.

Variant 5 shows an upward trend in the moisture content of the thermal material—amounting to 12% in the 8th year of operation, and in the case of variants 1 and 2, the moisture content of mineral wool is at the same level at a max. of 4%. For variant 5, the moisture content peaks at 5% throughout the analyzed period. In the case of moisture content in the gypsum board, the use of a moisture barrier of PE film—whether under the grid, above the grid or by filling the air gap with material—does not affect the amount of moisture appearing in the board, and is 0.44–0.80%. In variants 4 and 5, the amplitude of changes in the moisture content of the gypsum board is greater, namely 0.3–0.85% (variant 4) and 0.3–0.92% (variant 5). As can be seen from the analysis, the moisture content of gypsum plasterboard does not exceed about 0.9%, which does not have a degrading effect on the operation of the partition (except for variant 5). In the case of thermal material, moisture as low as 4% reduces the thermal insulation of the partition by 50%, and at 12%, causes its thermal insulation parameters to drop to 20%. It should be noted here that in variant 5, the moisture content of wool never falls below 5% as early as the second year of operation of the partition. In variants 1, 2, 3 and 4, moisture can build up to 4%, but in summer periods the moisture level falls below 2%. This kind of wetting, especially short-term, does not damage the structure of the wool, and should not result in the permanent deterioration of its thermal or strength properties. In the case of variant 5, the dampness is above 5% from the 2nd year of operation onwards, that is, the wool is soggy and wet, which indicates the possibility of layer degradation and mycological changes.

## 4. Discussion

Many building are characterized by insufficient air exchange, which may result in the symptoms of sick building syndrome (SBS). A large number of existing buildings are equipped with natural ventilation, but whose effectiveness is reduced by energy-saving activities [71].

Article [72] studies ventilation driven by thermal buoyancy in the air cavity of inclined roofs. The influence of air cavity design and roof inclination on the airflow is investigated. Combinations of different roof inclinations, air cavity heights and applied heating power on the air cavity top surface were examined. The study showed that increased air cavity height led to increased airflow and decreased surface temperatures in the air cavity. Increased roof inclination and heating power applied to the roofing also increased the airflow.

Thanks to the numerical simulation of temperature fields and other parameters of modelled partitions, it is possible to obtain a lot of information about their operation in the long term [62,73]. The analysed partition meets the current thermal standards of buildings. The obtained results of simulation calculations show no influence of the position of PE foil (acting as a vapour barrier, or type of “vapour retarder”) against the plasterboard fixing grate on the moisture condition of thermal insulation materials or plasterboard used. This is proven by the results for variants 1 and 2 of the calculations. Moreover, the possible sagging of “rock” mineral wool boards and their contact with the surface of PE foil does not affect moisture content of other built-in materials, as shown in variant 3 of the calculations. When the natural ventilation of roof airspace works (correctly), lack of PE foil does not significantly affect the moisture condition of thermal insulation materials or plasterboards, as shown in variant 4 of the calculation. Only the time of the highest moisture content of glass mineral wool is shifted. Lack of a vapour barrier with simultaneous lack of ventilation of roof airspace creates a risk of a gradual increase in moisture content of thermal insulation materials. In addition, conditions for the development of mould appear on the surface of plasterboards. The occurrence of these phenomena is indicated by variant 5 of the calculations. In double-sided ventilated flat roofs, when there is no water vapour concentration, no vapour barrier is required. Above wet rooms (water vapour pressure above 2150 Pa, i.e., temperature inside ≥27 °C and approx. 20 h of hot water evaporation), if it is impossible to use a double-sided ventilated flat roof with a well-ventilated airspace, a solution with an appropriate vapour barrier is used, the type of which depends on the value of diffusion resistance of subsequent layers. For the analysed case, the water vapour pressure did not exceed the given limit at the given temperatures. The highest temperature was 25 °C and corresponded to 70% relative humidity. Even with extreme environmental conditions, with active ventilation (gravitational or mechanical), during the simulated period, the problem of condensation of an excessive amount of water vapour will not occur regardless of whether or not the vapour barrier layers are arranged on the inside of wet rooms.

## 5. Conclusions

The “sick” building syndrome (SBS) or mycological changes appearing on walls and ceilings are largely affected by the excessive amount of water generated in buildings. Moistened materials contribute to the destruction and faster wear of structures. Properly made ventilation and modelled partition layers protect roof structures against moisture and related further damage.

The numerical design simulation showed that flat roofs containing air spaces should be treated differently to pitched roofs containing small, ventilated spaces in their layers. In the case of flat roofs, it is not always necessary to use flexible waterproofing materials in ventilated partitions to protect their thermal insulation against moisture.

For variants 1–3 (Figure 3, Figure 4 and Figure 5), the moisture level for mineral wool during the analysed period ranged from 1.7% to 4%; for variant 4 (Figure 6), it ranged from 2.1% to 4.7%; while for variant 5 (Figure 7), moisture content for mineral wool increased throughout the research period, starting from 2% in winter to 12% (maximum value last year in summer).

For variants 1–3 (Figure 3, Figure 4 and Figure 5), the moisture level for plasterboard ranged from 0.44% to 0.80%; for variant 4 (Figure 6), it ranged from 0.3% to 0.85%; while for variant 5 (Figure 7), moisture content for plasterboard fluctuated throughout the research period from 0.3% in winter to 0.92% (maximum value last year in summer).

For variants 1–3 (Figure 3, Figure 4 and Figure 5), mould spots appeared at a minimum relative humidity of 41% and a temperature of 18 °C; and for variant 4, mould spots appeared at a minimum relative humidity of 30% and a temperature of 18 °C. For variants 1–4 (Figure 3, Figure 4, Figure 5 and Figure 6), there are no critical values in the isopleth (Figure 11 and Figure 14), i.e., the point where the curves intersect with the area of points. However, for variant 5 (Figure 7), the risk of mould development appeared at a minimum relative humidity of 78% and a temperature of approx. 24 °C (Figure 17).

The numerical analysis with the assumptions of extreme parameters showed that the problem with moisture will appear in variant 5, i.e., in the absence of PE foil in the system of layers and in the absence of ventilation of the flat roof.

The numerical analysis showed that proper multi-variant modelling reduces the energy loss for heating buildings and eliminates the problem of damage to thermal and finishing materials. Additionally, it should be noted that the use of a flexible waterproofing material in the layers of a double-sided ventilated flat roof is not necessary, even if humidity in the rooms under the roof exceeds 70% and ventilation is carried out by gravity.

The manuscript presents a variety of solutions aimed at designers to protect a ventilated roof from moisture penetrating the thermal insulation and ceiling (gypsum board) layers. An analysis of the validity of the use of a ventilated waterproofing layer in a multilayer partition is presented, with a simulation of the work of such a layer or its absence in the partition over 8 years. In the previously available literature, there has been little research or analysis on the validity of the use of a waterproofing layer placed in different locations of the ventilated ceiling, or evaluation of the variation of moisture in individual layers.

## Figures and Tables

**Table 1:** Water vapour content in the air with the maximum humidity depending on temperature [42].

Humid Air Temperature RH = 100% [°C]	Water Vapour Content [g/m^3^]	Humid Air Temperature RH = 100% [°C]	Water Vapour Content [g/m^3^]	Humid Air Temperature RH = 100% [°C]	Water Vapour Content [g/m^3^]
−20	0.9	6	7.3	21	18.4
−15	1.4	8	8.3	22	19.5
−10	2.1	10	9.4	23	20.6
−8	2.5	12	10.7	24	21.8
−6	3.0	14	12.1	25	23.1
−4	3.5	16	13.7	26	24.4
−2	4.1	17	14.5	28	27.2
0	4.8	18	15.4	30	30.4
2	5.6	19	16.3	40	51.1
4	6.4	20	17.3	50	82.3

**Table 2:** Simulation of the source of humidity and the amount of water vapour—author’s summary [58].

Source of Humidity	Water Vapour Emission in 24 h [g]	Amount of Water Vapour Reaching the Attic in 24 h [g]	
		with 10× Exchange	with 25× Exchange
Respiration and sweat evaporation	5000	3500	2500
Use of a residential building	13,255	9279	9279
Potted plants (5–7 plants per apartment)	500	350	250
Total	18,755 g	13,129 g	12,029 g

**Table 3:** Total amount of water vapour reaching the attic depending on the adopted air exchange [58].

	Amount of Water Vapour Reaching the Attic in 24 h at 80% Relative Humidity and Air Temperature 20 °C [g]	Amount of Water Vapour Reaching the Attic in 24 h Generated in a Residential Building [g]	Total [g]
10× exchange	40,689.6	13,129.0	53,818.6
25× exchange	72,660.0	12,029.0	84,689.0

**Table 4:** Relative humidity in a model building depending on air exchange [58].

	Absolute Humidity [g/m^3^]	Saturation State at 20 °C [g/m^3^]	Relative Humidity [%]
10× exchange	18.31	17.30	100% supersaturation state
25× exchange	16.13		93%

**Table 5:** Flexible waterproofing products used as initial covering layers [53].

ICF—Initial Covering Foils (Low Vapour-Permeable)		ICM—Initial Covering Membranes (Highly Vapour-Permeable)	
Vapour-tight	Vapour-permeable	Light	Screens

## Data Availability

Not applicable.
